# Supplementary material for: Hepatoprotective Activity of Lignin-Derived Polyphenols Dereplicated Using High-Resolution Mass Spectrometry, In Vivo Experiments, and Deep Learning
Source: Int J Mol Sci. 2022 Dec 16;23(24):16025. doi: 10.3390/ijms232416025 (PMC9781348; doi:10.3390/ijms232416025)
Supplement: Supplementary file 1 [file ijms-23-16025-s001.zip › ijms-2059976-supplementary.pdf]

Supporting Information

Hepatoprotective Activity of Lignin-Derived Polyphenols Dereplicated Using High-Resolution Mass Spectrometry, In Vivo Experiments, and Deep Learning

Alexey Orlov [1], Savva Semenov [1,2], Gleb Rukhovich [1], Anastasia Sarycheva [1], Oxana Kovaleva [1], Alexander Semenov [3], Elena Ermakova [3], Ekaterina Gubareva [3], Anna E. Bugrova [4], Alexey Kononikhin [1], Elena I. Fedoros [3], Evgeny Nikolaev [1,*] and Alexander Zherebker [1,*]

[1] Skolkovo Institute of Science and Technology, 30 Bolshoy Boulevard bld. 1, 121205 Moscow, Russia

[2] Moscow Institute of Physics and Technology, 141701 Moscow, Russia

[3] N.N. Petrov National Medical Research Center of Oncology, 68 Leningradskaya str, 197758 Saint Petersburg, Russia

[4] Emanuel Institute for Biochemical Physics, Russian Academy of Sciences, 119334 Moscow, Russia

***** Correspondence: e.nikolaev@skoltech.ru (E.N.); a.zherebker@skoltech.ru (A.Z.)

This file includes

Pages: 15

Figures S1 to S8

Tables: S3 to S8

Legend for Tables S1-S2

Other Supporting Information for this manuscript:

Table S1, S2 (separate xlsx file)

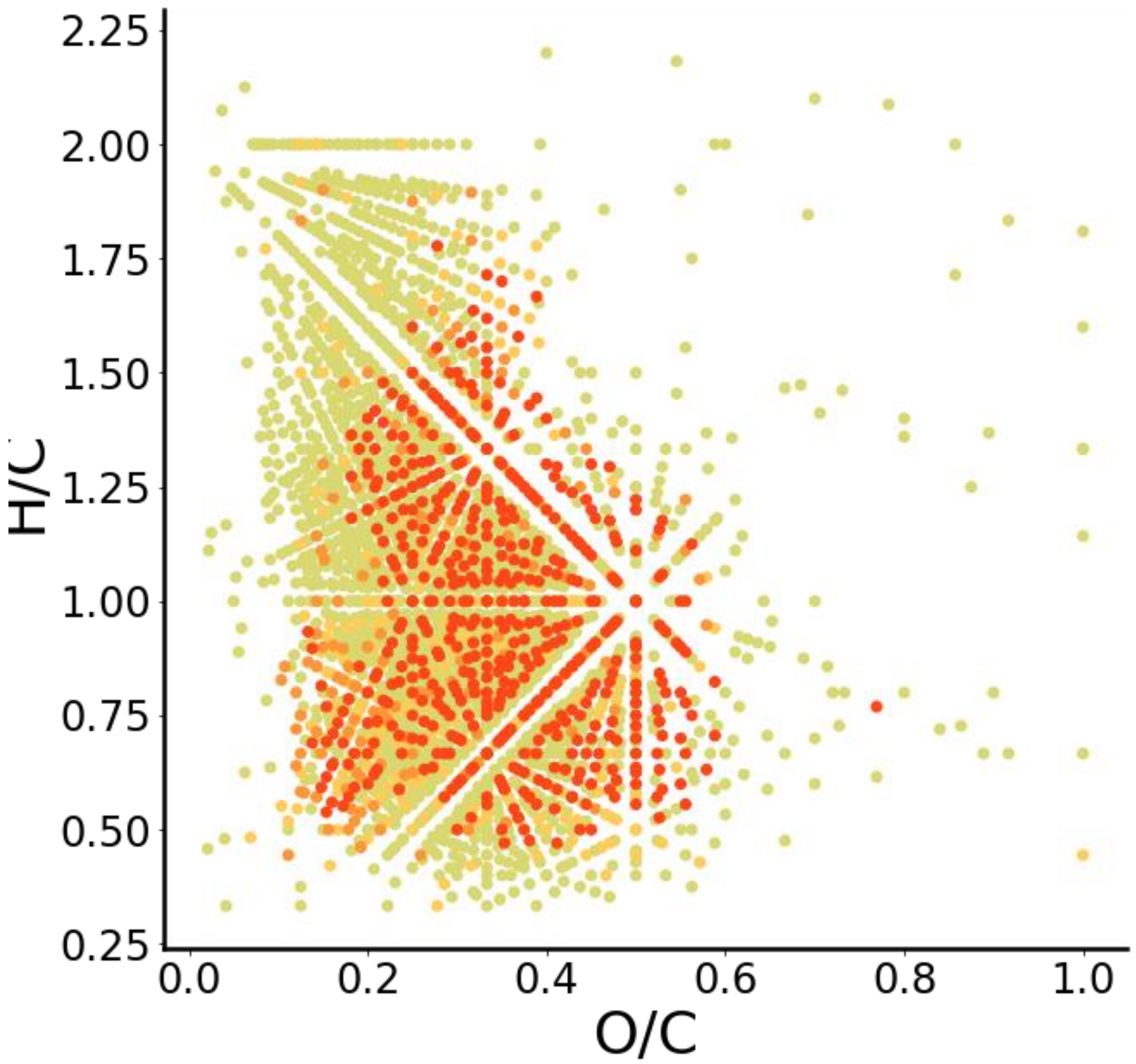

Figure S1. Van Krevelen diagrams for BP-Cx-1. The color scheme is related to the number of H/D exchanges in NaOD: pale green – 0, yellow – 1, orange – 2, red – ≥3.

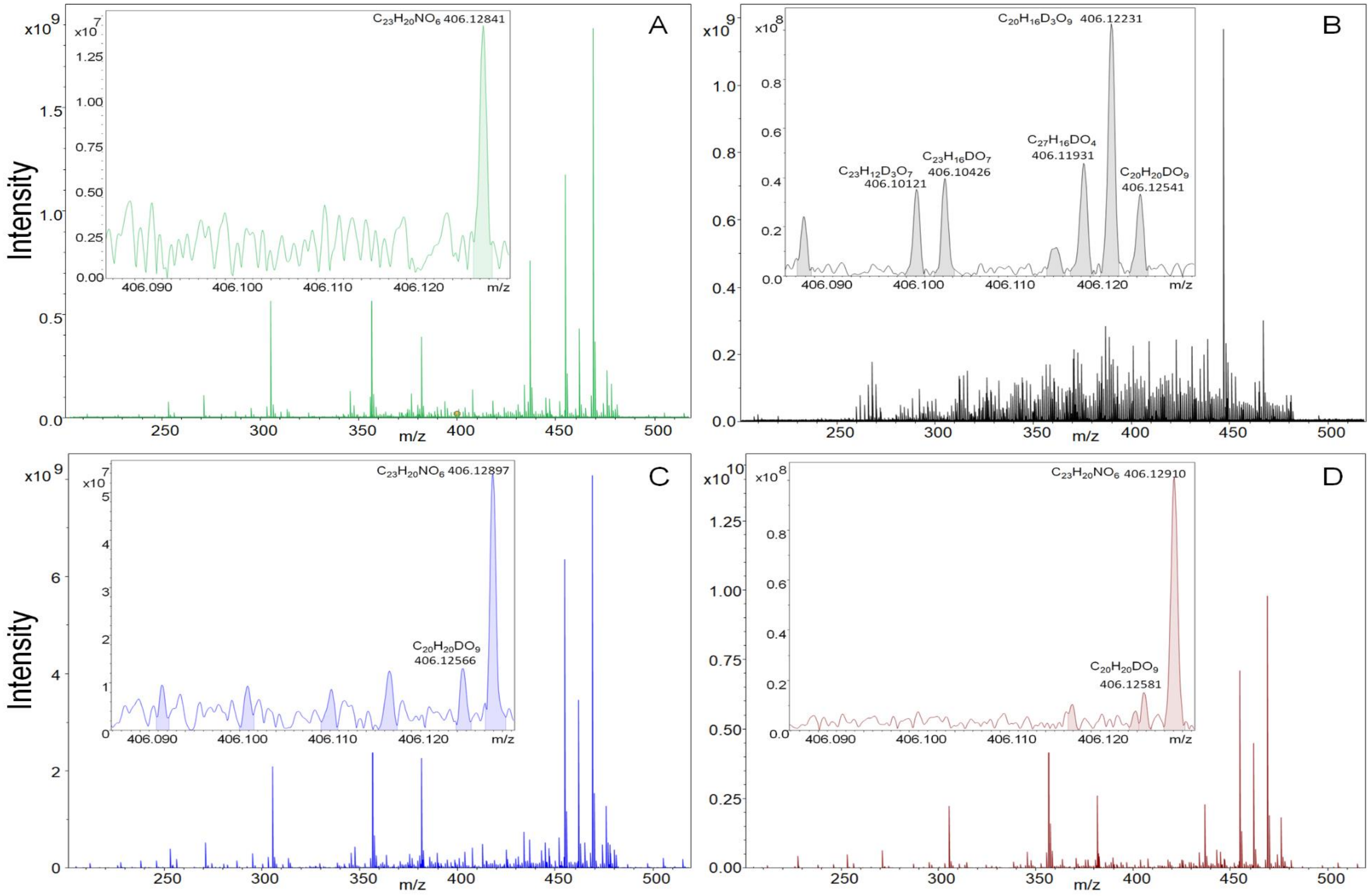

Figure S2. Q-isolated FTICR mass-spectra of A) control liver extract, B) D-labeled BP-Cx-1, C,D) Liver extract after intravenous and gavage administration of D-labeled BP-Cx-1, respectively. Insets include magnified mass-spectra region with clear D-containing ion, which was absent in control sample

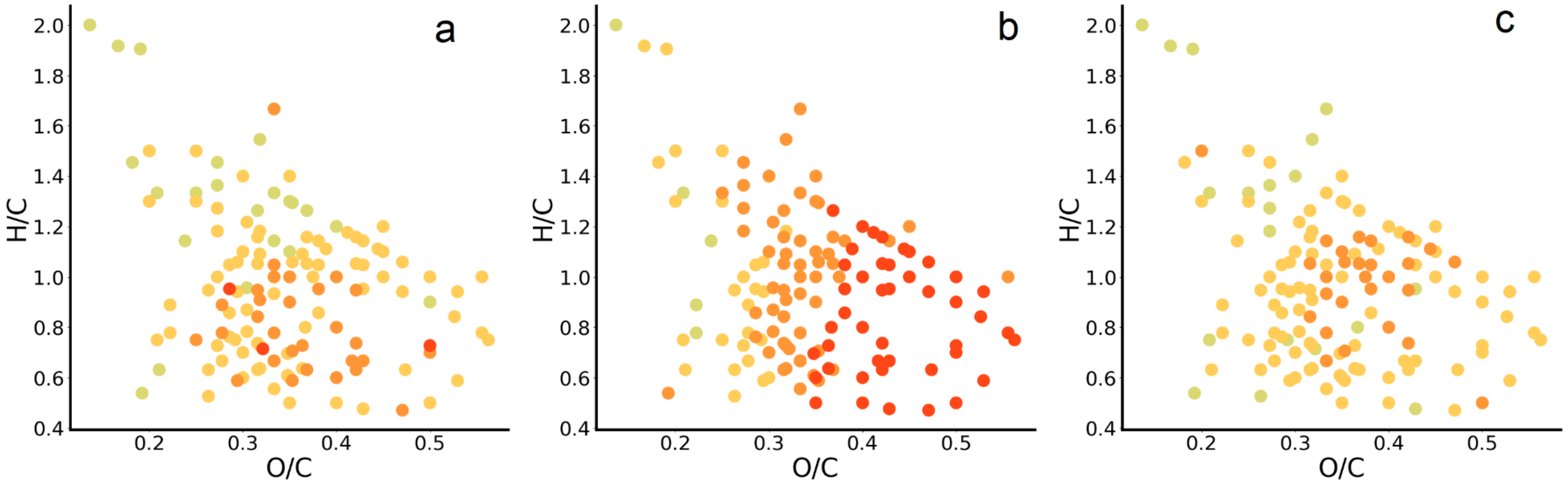

Figure S3. Van Krevelen diagrams for 133 molecular components of BP-Cx-1 detected in liver. The color scheme is related to the number of detected structural moieties (**a** – carbonyl, **b** – carboxyl, **c** – aromatic rings): pale green – 0, yellow – 1, orange – 2, red – ≥3.

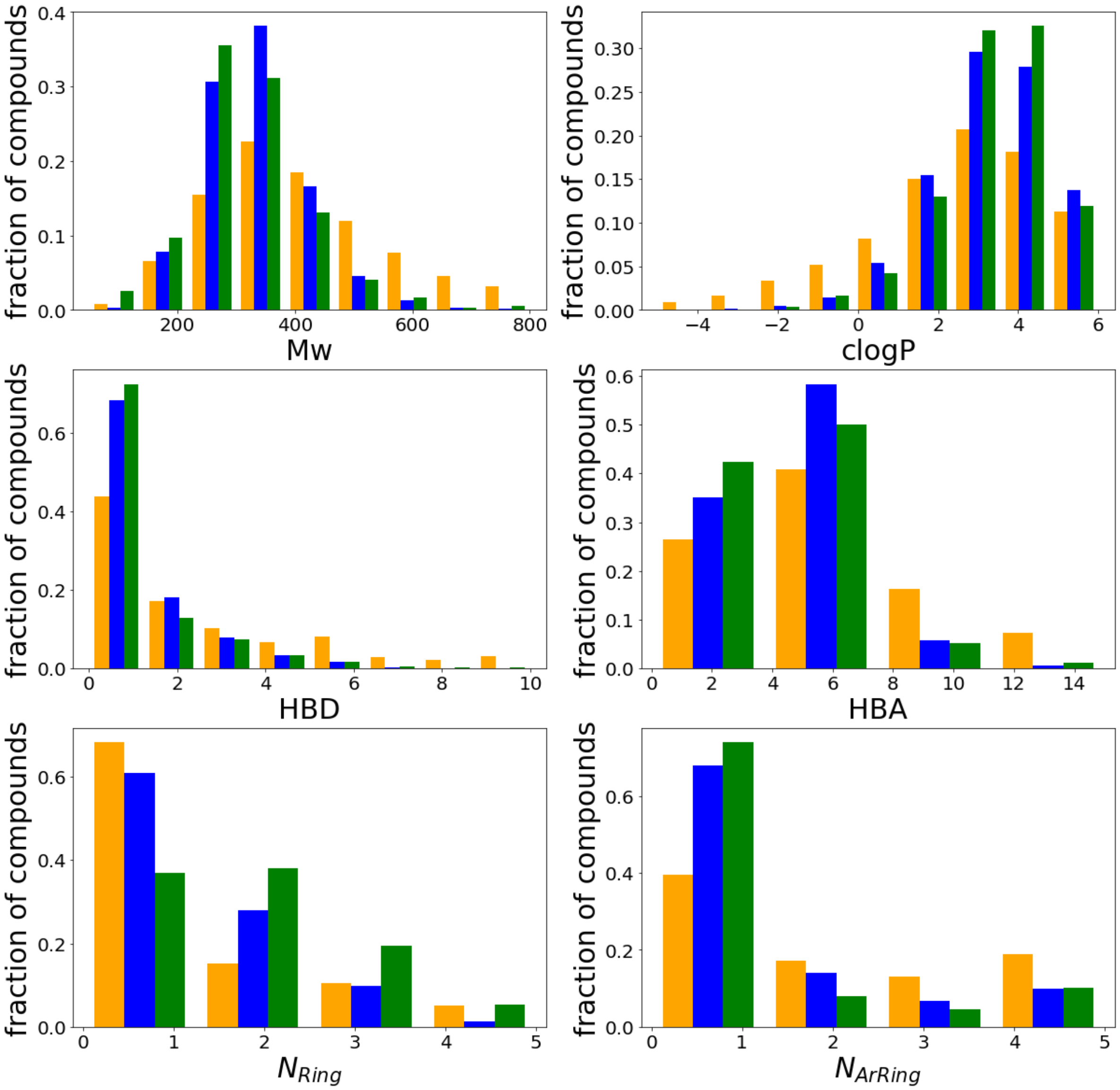

Figure S4. Distribution of molecular descriptors of compounds from CoCoNut $C_xH_yO_z$ subset (orange), ChEMBL compounds active against Nrf2 (green), generated compounds (blue). a Molecular weight (Mw), b calculated lipophilicity (clogP), c number of hydrogen bond donors (HBD), d number of hydrogen bond acceptors (HBA), e number of aliphatic rings(N_{Ring}), f number of aromatic rings (N_{ArRing}).

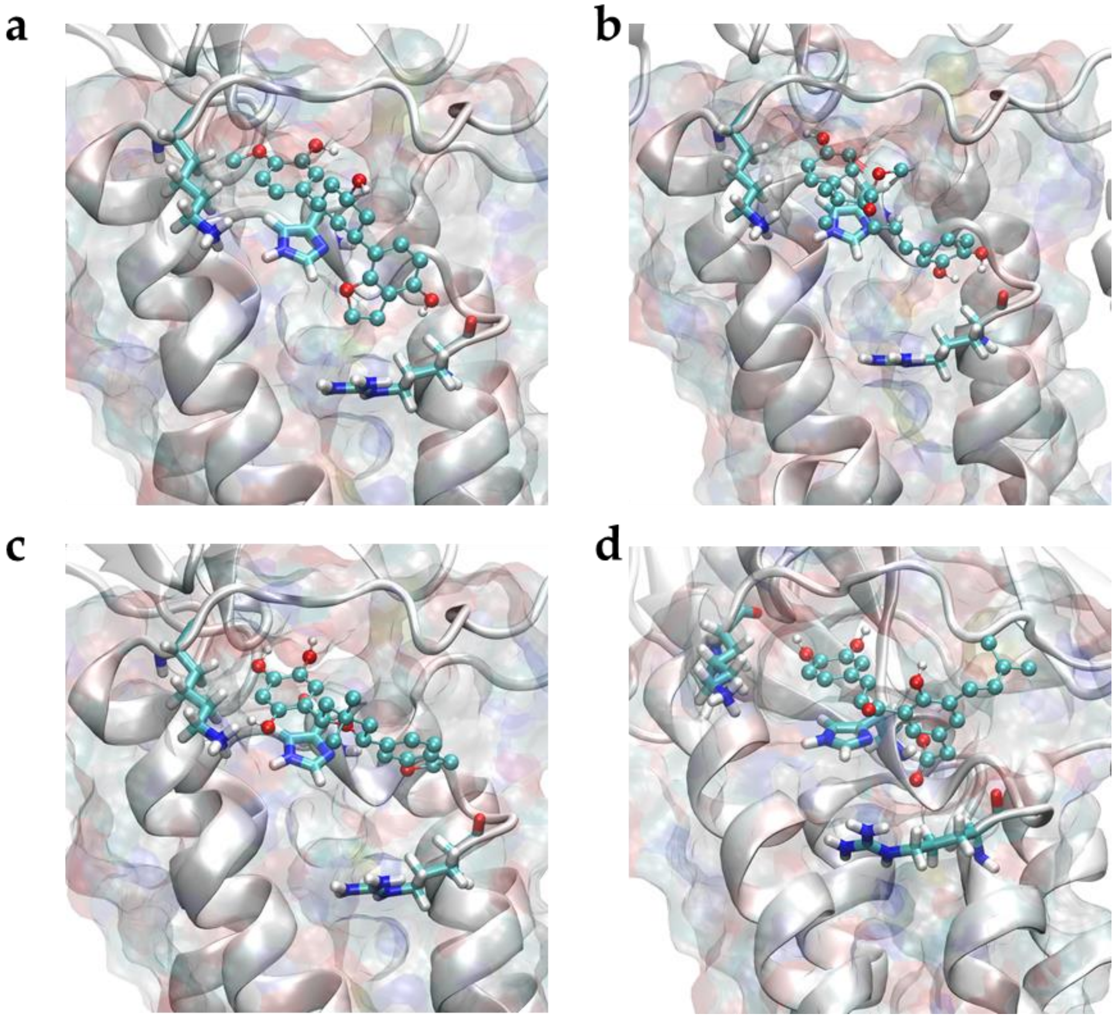

Figure S5. Top scoring binding poses. a compound 1, b compound 2, c compound 3, d compound 4

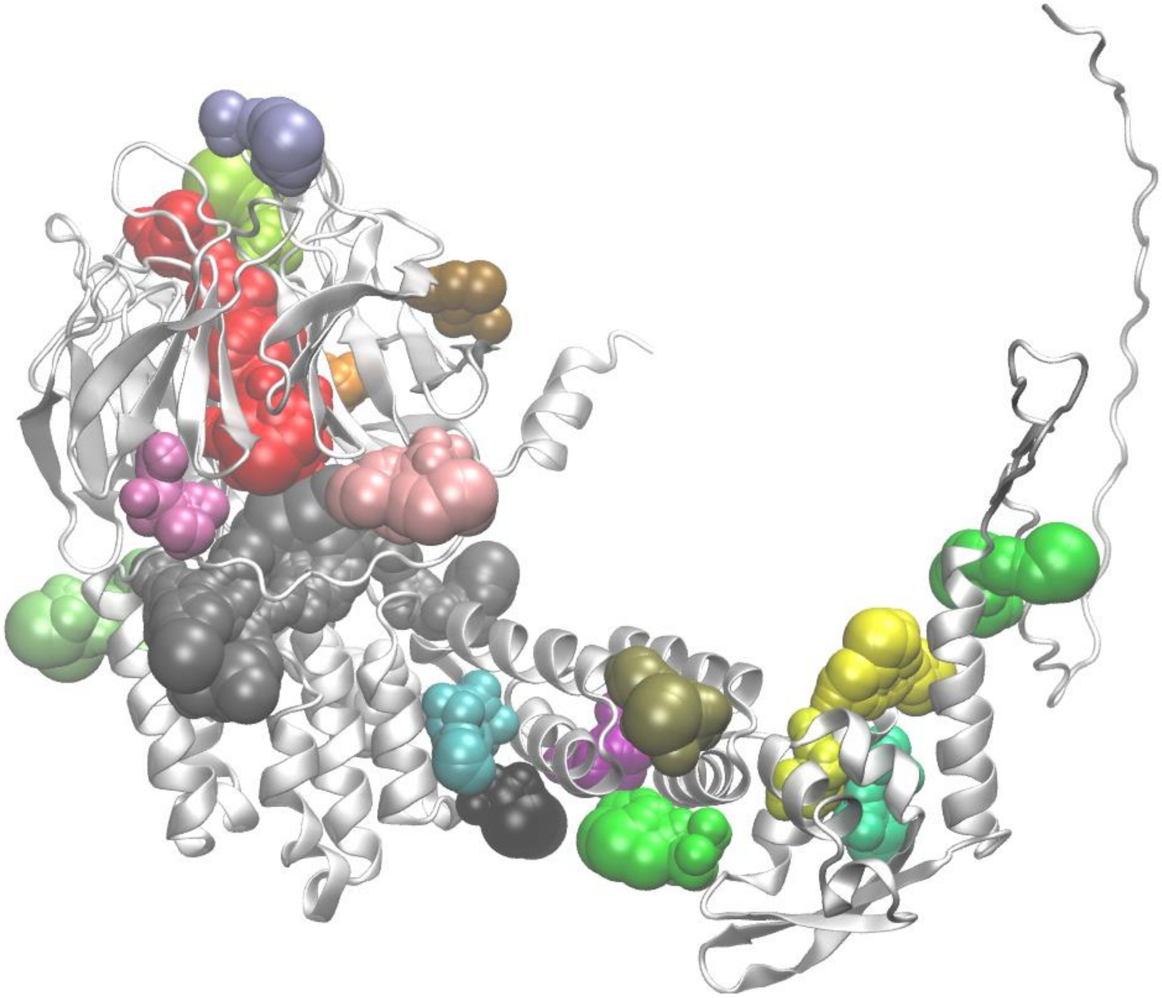

Figure S6. Cavities (shown as spheres) on the surface of a full-length KEAP1 model predicted by fpocket2.

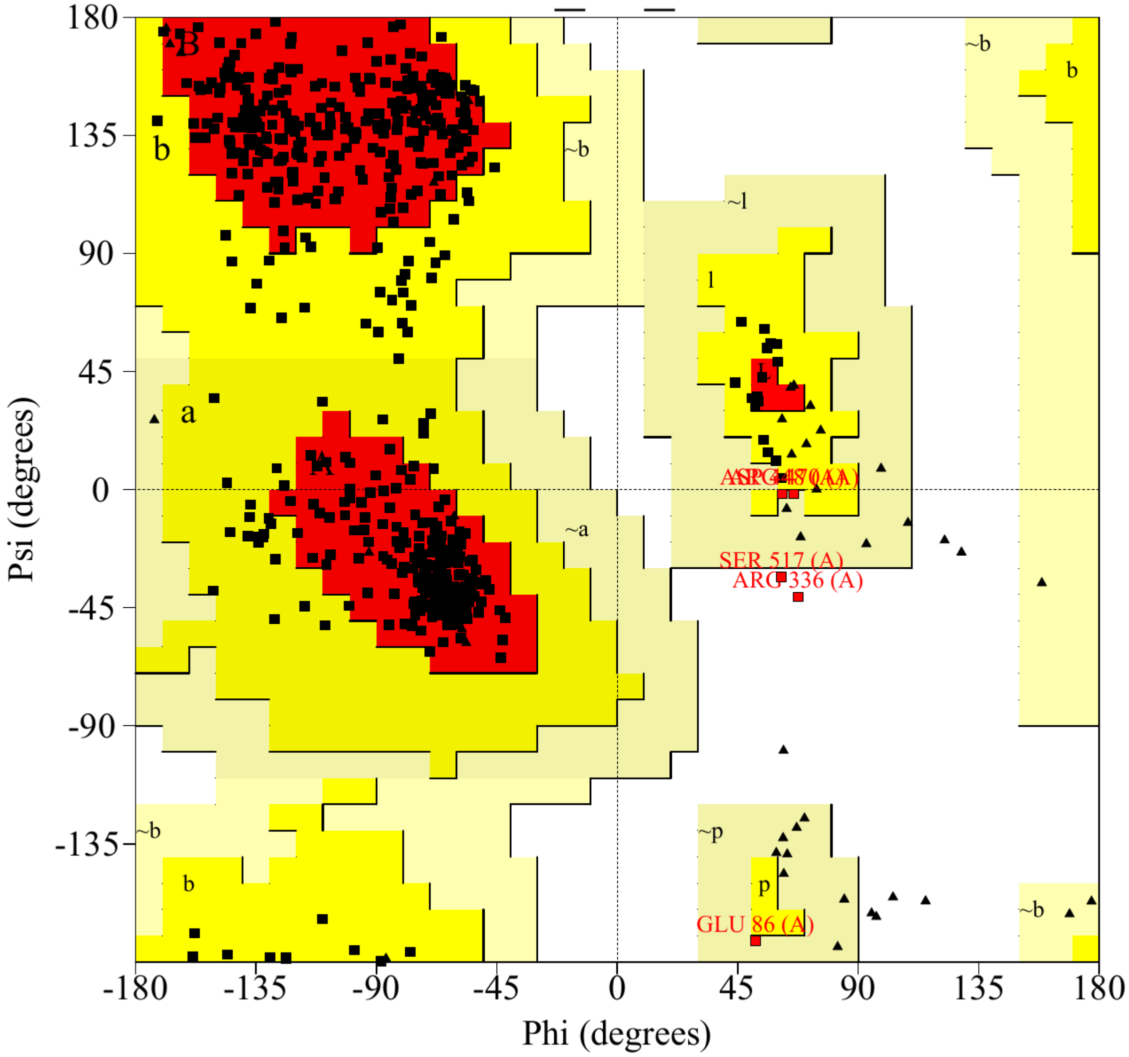

Figure S7. Screenshot of a Ramachadran plot obtained with PROCHECK 2.3. Residues from disallowed regions (Arg470, Glu86, Ser517, Arg336, Asp448) are labeled.

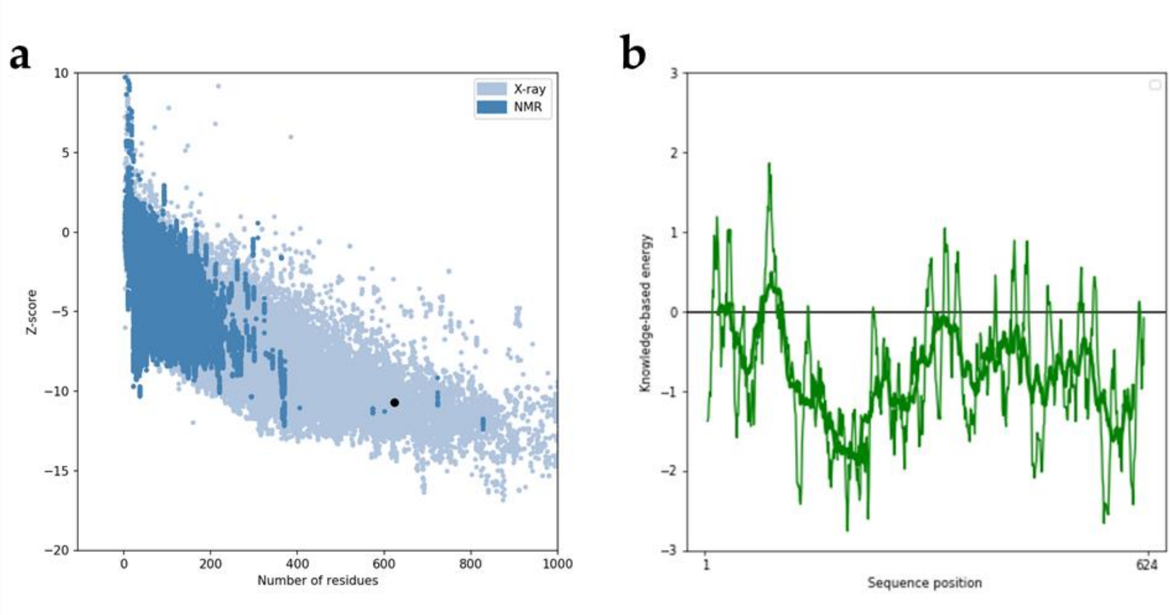

Figure S8. a. Screenshot of ProSA-web z-score plot for the full-length KEAP1 model. The z-score (-10.71) indicates overall model quality. Its value is displayed in a plot that contains the z-scores of all experimentally determined protein chains (X-ray (pale blue), NMR (blue)) in current PDB. **b.** Screenshot of ProSA-web plot of residue scores for the full-length KEAP1 model. This plot shows local model quality by plotting energies as a function of amino acid sequence position i. Smoothing window of 40 residues was used.

Tables S1. Results on H/D exchange of BP-Cx-1 in NaOD as measured by FTICR MS

Tables S2. BP-Cx-1 components determined in liver with structural features as determinedby FTICR MS and isotope tagging

Tables S3. Number-averaged values of FTICR MS-derived data on BP-Cx-1 fractions

Fraction	Mn	$(AI_{con})n$	DBEn	(H/C)n	(O/C)n
MeOH100	459.44	0.45	13.01	1.08	0.31
MeOH75	527.59	0.57	17.70	0.87	0.31
MeOH50	492.33	0.59	17.58	0.83	0.30
MeOH25	438.06	0.48	14.13	1.02	0.27

Tables S4. Contribution of AI_{con}-based classes of BP-Cx-1 fractions obtained by FTICR MS

Fraction	NumForm	N-containing	Aliphatics	unsaturated (O/C<0.5)	unsataturated (O/C≥0.5)	aromatic (O/C<0.5)	aromatic (O/C≥0.5)	condensed (O/C<0.5)	condensed (O/C≥0.5)
MeOH100	5927	0.85%	1.67%	30.98%	0.18%	56.75%	0.71%	8.64%	0.21%
MeOH75	4379	0.03%	0.05%	15.59%	0.02%	67.32%	0.11%	16.81%	0.06%
MeOH50	4546	0.08%	0.85%	6.88%	0.07%	57.40%	0.16%	34.52%	0.03%
MeOH25	3676	0.31%	1.55%	9.82%	0.20%	48.80%	0.31%	38.96%	0.05%

Tables S5. Body weight of female mice with induced NAFLD accompanied by type 2 diabetes, g, (M±m)

**Group**	**Week**							
	**0**	**1***	**2**	**3****	**4*****	**5**	**6**	**7**
1	26,3±0,6	26±0,4	26,3±0,5	26,6±0,5	26,4±0,5	27,1±0,5	27,3±0,5	25,9±0,4
2	25,7±0,4	25,6±0,5	25,5±0,3	*22,8±0,3*	*21±0,5*	*21,4±0,9*	*22±1,0*	*20,2±0,8*
3	25,9±0,7	25,5±0,8	25,9±0,6	*21,8±0,6*	*21,4±0,7*	*22,2±0,9*	*21,6±0,9*	*20,9±1,1*
4	26,3±0,5	25,8±0,6	25,7±0,5	*22,8±0,8*	*21,6±1,0*	*21,5±0,6*	*20,6±0,7*	*21,2±0,8*

Comments:
*Start of Начало HFD and HFD
**Streptozotocin administration
***Start of BP-Cx-1 or its fraction administration
Italic: significant deviation with group 1
Bold: significant deviation of groups 3-4 with group 2

Table S6. Fluid and food intake in female mice with induced NAFLD accompanied by type 2 diabetes

**Group**	**Week**		**0**	**1**	**2***	**3**	**4**	**5**
**1**	Fluid intake, ml/mouse/day		3,4±0,2	3,4±0,1	3,5±0,2	3,7±0,1	3,9±0,1	3,9±0,1
	Intake of standard feed, g/mouse/day		2,8±0,5	2,9±0,3	2,9±0,4	3,4±0,2	3,5±0,1	3,5±0,1
**2**	Fluid intake, ml/mouse/day		2,9±0,2	3±0,2	4,3±0,9	4,5±0,7	4,5±0,6	3,6±0,3
	Intake of solid feed, g/mouse/day	Total	2,2±0,1	-	*2,2±0,1*	*2±0,1*	*1,9±0,2*	*1,7±0,2*
		Standard feed	*0,8±0,1*	0,7±0,1	*0,7±0,1*	*0,5±0,1*	*0,5±0,1*	*0,4±0,1*
		Lard	1,5±0,1	-	1,5±0,1	1,5±0,1	1,4±0,1	1,3±0,1
**3**	Fluid intake, ml/mouse/day		2,6±0,2	2,7±0,2	3,9±0,8	3,8±0,7	3,6±0,6	2,6±0,2
	Intake of solid feed, g/mouse/day	Total	2,1±0,1	-	*2,1±0,1*	*1,8±0,1*	*1,8±0,1*	*1,7±0,1*
		Standard feed	*0,8±0,1*	0,7±0,1	*0,6±0,1*	*0,4±0,1*	*0,5±0,1*	*0,4±0,1*
		Lard	0,1±1,5	-	0,1±1,5	0,1±1,9	0,1±1,6	0,1±1,7
**4**	Fluid intake, ml/mouse/day		2,8±0,2	2,9±0,2	3,2±0,3	3,4±0,2	4,3±0,5	4,4±0,7
	Intake of solid feed, g/mouse/day	Total	2,1±0,1	-	*2,1±0,1*	*2,1±0,1*	*2,2±0,1*	***2,2±0,1***
		Standard feed	*0,9±0,1*	0,8±0,1	*0,6±0,1*	*0,6±0,1*	*0,8±0,1*	***0,9±0,1***
		Lard	1,4±0,1	-	1,4±0,1	1,5±0,1	1,3±0,1	1,4±0,1

Comments:
* Streptozotocin administration
Italic: significant deviation with group 1
Bold: significant deviation of groups 3-4 with group 2

Table S7. Results of a final clinical blood analysis of female mice with induced NAFLD accompanied by type 2 diabetes

**Groups**	**Leukocytes**	**Lymphocytes**	**Monocytes**	**Granulocytes**	**Erythrocytes**	**Hemoglobin**	**Hematocrits**	**Thrombocytes**	**MCHC**
	WBC 10^9/L	Lymph# 10^9/L	Mon# 10^9/L	Gran# 10^9/L	RBC 10^12/L	HGB g/L	HCT %	PLT 10^9/L	MCHC, g/L
**1**	3,5±0,2	2,3±0,1	0,1±0,02	1,1±0,1	10,3±0,1	150,2±1,5	46,4±0,3	968,4±62,1	323,4±1,8
**2**	7,2±1,6	2,3±0,6	0,3±0,1	3,9±1,3	*11,2±0,1*	165,3±8	*50,5±0,9*	1247,8±124,7	326,8±14,5
**3**	12,5±6,2	4,2±0,6	0,5±0,3	7,8±5,4	**10,6±0,2**	152,8±2,1	**46,9±0,7**	1007,8±101,2	325,3±3,5
**4**	4,3±0,5	2,2±0,3	0,1±0,02	1,9±0,2	**10,3±0,1**	152,4±4,4	**45,9±0,3**	1140,4±72,2	331,4±8,2

Comments:
Italic: significant deviation with group 1
Bold: significant deviation of groups 3-4 with group 2

Table S8. The most frequent scaffolds in a CoCoNut subset (standardized $C_xH_yO_z$ compounds, CoCoNut $C_xH_yO_z$), a subset of ChEMBL compounds interfering with an Nrf2 pathway (ChEMBL Nrf2), and generated compounds.

CoCoNut $C_xH_yO_z$		ChEMBL Nrf2		generated compounds	
Scaffolds					
Scaffold	%	Scaffold	%	Scaffold	%
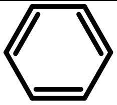	3.3	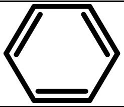	4.9	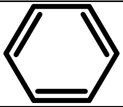	6.3
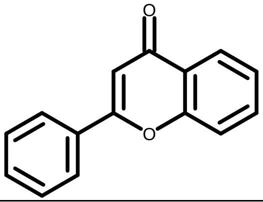	1.0	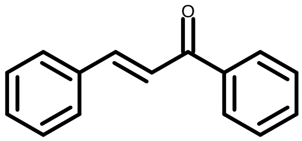	3.8	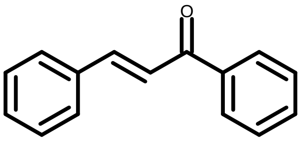	5.2
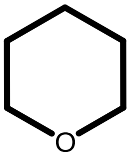	0.68	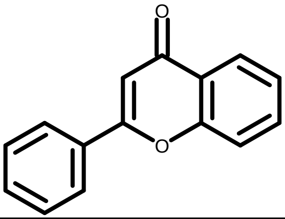	1.9	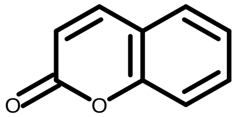	1.0
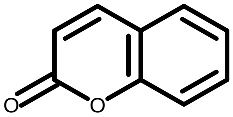	0.67	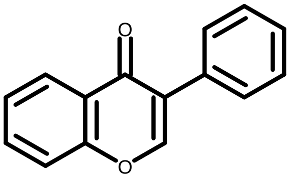	1.6	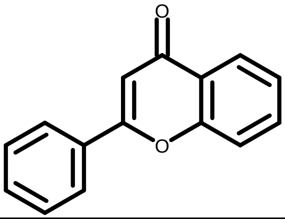	1.0
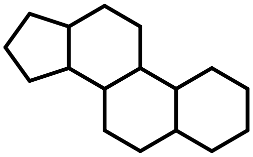	0.58	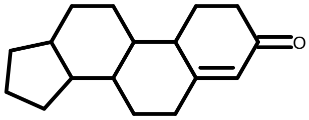	1.5	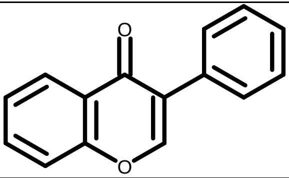	0.6
